# Meta-Analysis-Based Comparison of Annual Fall Risk between Older Adults with Alzheimer’s Disease and Mild Cognitive Impairment

**DOI:** 10.20900/agmr20240002

**Published:** 2024-03-20

**Authors:** Caroline Simpkins, Sara Mahmoudzadeh Khalili, Feng Yang

**Affiliations:** 1 Department of Kinesiology and Health, Georgia State University, Atlanta, GA 30303, USA; † These authors contributed equally

**Keywords:** dementia, fall prevention, faller, number of falls

## Abstract

**Background:**

Falls are a primary cause of injuries and hospitalization in older adults. It has been reported that cognitive impairments and dementia can increase fall risk in the older population; however, it remains unknown if fall risk differs among subgroups of dementia. This meta-analysis summarized previous studies reporting the annual fall risk of people with Alzheimer’s disease (AD) or mild cognitive impairment (MCI) and compared the fall risk between these two groups of people with dementia.

**Methods:**

Thirty-five studies enrolling 7844 older adults with AD or MCI were included. The annual fall prevalence and average number of falls of the included studies were meta-analyzed and compared by random-effects models with inverse variance weights.

**Results:**

The annual fall prevalence in people with AD (43.55%) was significantly higher than MCI (35.26%, *p* < 0.001). A *χ*^2^ test indicated that the pooled fall prevalence is significantly higher in people with AD than MCI *χ*^2^ = 158.403, *p* < 0.001). Additionally, the yearly average number of falls in AD was higher than in MCI (1.30 vs 0.77 falls/person).

**Conclusions:**

The results showed that older people with AD experience a higher annual fall prevalence with a larger number of falls than older adults with MCI. The results suggested that the fall risk measurements should be reported separately between people with AD and MCI. The findings could provide preliminary guidance for the identification of individuals with dementia who experience a high fall risk.

## INTRODUCTION

Falls are a serious health threat among older adults [1]. Approximately 26.5% of older adults fall annually with catastrophic consequences including physical injuries or even death [2,3], limited independence, institutionalization, and costly economic burden [4,5]. Dementia heightens the risk of falls in older adults [6] and roughly 80% of dementia patients fall annually [7]. A higher risk of falls in older adults with dementia and cognitive impairment could be due to the inability to clear obstacles, poor balance (especially during dual tasks), difficulty in spatial navigation, and an increased mobility dysfunction [8]. Currently, 50 million people are affected by dementia, and it is predicted that the number of people with dementia will reach 150 million globally by 2050 [9]. Alzheimer’s disease (AD) is the most common cause of dementia in older individuals [10] and affects approximately 10%–30% of older adults [11]. Furthermore, mild cognitive impairment (MCI) encompasses the early stage of cognitive disorders [12], and 33.6%–90% of individuals with MCI are estimated to progress to AD [13]. AD and MCI significantly elevate the fall risk in older adults, as it has been reported that 59% of older adults with MCI fall at least once a year [14], and AD raises the fall risk by about 70% among older adults [15]. The risk of fall-related injuries is also higher in people with AD or MCI than in their cognitively intact peers [16]. As the population ages and life expectancy extends, the risk of falls and dementia upsurge [17]. For example, 21.3% of adults aged 85 years or older fall quarterly [18]. Additionally, about 6.2 million older Americans are affected by AD, and the number of new AD cases is projected to double in the U.S. by the year 2050 [19]. Clearly, falls are a serious public concern due to the commingling effect of dementia and the aging-induced fall risk. It is, therefore, necessary to understand the magnitude of falls for robust estimates of the healthcare costs related to falls and their reduction for people with AD or MCI.

Preventing falls for people with AD or MCI is critically important, yet a recent meta-analysis concluded that the effect of fall prevention interventions on falls in cognitively impaired individuals remains unclear [20]. One essential condition for developing fall prevention strategies is to thoroughly grasp how dementia status affects the fall risk in older adults. The annual fall prevalence and number of falls have been used as common metrics of fall risk in the literature. Some past studies merged the AD and MCI groups when reporting the annual fall prevalence or number of falls [7,21]. This approach could be problematic as it assumes that the fall risk is similar in these two subgroups of people with dementia. Additionally, considering that the degree of cognitive impairment in people with AD and MCI is dissimilar, fall risk could be different between these two groups and should be reported separately. For example, if the fall risk differs between AD and MCI, a combined fall risk could overestimate the fall risk in one group while underestimating it in the other group. This fall risk miscalculation could lead to an inefficient use of limited healthcare resources allocated to fall prevention for people with dementia or create barriers to developing effective fall prevention programs for these populations. As the number of older adults affected by AD or MCI continues to increase at an alarming rate [17], an accurate estimation of the potential fall risk in either group is imperative.

Very few studies have compared the fall risk in older adults with AD and MCI and such studies have also presented inconsistent findings. For instance, one study reported that the annual fall prevalence in older adults with MCI (52.6%) is higher than AD (51.3%) [22], while another study suggested an opposing viewpoint that people with AD experience a higher annual fall prevalence (65.3%) compared to MCI (59.5%) [14]. These inconclusive findings could be due to the small sample sizes and diverse study conditions and settings. It is possible to benefit from meta-analyzing previously published studies that reported the fall prevalence and number of falls in these populations [14,22]. Over the past decades, numerous published articles either directly or indirectly documented the fall risk metrics for these two groups of individuals with dementia (AD vs MCI). Therefore, the conduction of such meta-analyses is feasible.

The primary purposes of this meta-analysis were (1) to summarize the related literature and derive the annual fall prevalence and number of falls for the two dementia subgroups (AD and MCI), and (2) to compare the annual fall prevalence and number of falls between people with AD and MCI. Given that people with AD experience more severe cognitive impairments than those with MCI [14] and cognition deficits are associated with falls [23], it was hypothesized that people with AD would exhibit a higher fall risk in comparison with MCI, as reflected by the higher annual fall prevalence and number of falls. The findings of this study will provide insight into how cognitive impairment interacts with fall risk in older adults.

## MATERIALS AND METHODS

### Research Question

The purpose of this study was twofold: (1) to meta-analyze the literature to separately derive the fall risk for individuals with AD or MCI as quantified by the annual fall prevalence and number of falls, and (2) to determine if the fall risk metrics differ between people with AD and MCI.

### Literature Search

A literature search was performed following the Preferred Reporting Items for Systematic Reviews and Meta-Analyses (PRISMA) guidelines. The search was conducted in APA PSYCINFO, CINAHL, Google Scholar, MEDLINE, and PUBMED from their inception to February 2024. Search terms were developed for each database with the assistance of a university librarian and included combinations of the terms “Alzheimer’s”, “mild cognitive impairment”, “accidental falls”, and “falls” (Appendix 1). The search results were filtered to include only peer-reviewed articles published in English. No date or region restrictions were applied to the search.

### Study Selection and Eligibility Criteria

In this analysis, people with AD or MCI represented the population (P), and the two outcomes (O) of annual fall prevalence and number of falls were compared (C) between these two groups. Following duplicate removal, studies were screened independently by two authors (CS & SK) based on the following inclusion criteria: the study (1) was conducted among older adults with AD or MCI with an average age ≥ 65 years; (2) reported the number of fallers or falls over a 12-month tracking duration (either retrospectively or prospectively); and (3) was published in a peer-reviewed English journal. Studies were excluded if they (1) reported falls in people with dementia not caused by AD or MCI (e.g., Parkinson’s disease, Lewy body dementia, etc.); (2) were published as an abstract to safeguard the rigor of the meta-analysis; (3) used a fall tracking duration that was not 12 months; (4) only reported the fall prevalence or number of falls after an intervention; (5) merged people with AD and MCI into one group; and 6) were qualitative studies. No additional restrictions were placed on the study design of the considered articles. Any conflicts regarding study eligibility were resolved by a third author (FY).

### Study Assessment and Data Retrieval

The quality and certainty of the evidence were mainly assessed at the outcome level with the Grading of Recommendations, Assessment, Development, and Evaluation (GRADE) approach [24]. Five items (risk of bias, inconsistency, indirectness, imprecision, and publication bias) were scored as “not serious”, “serious”, or “very serious”. Based on this evaluation, the quality of evidence of each outcome was assessed as very low, low, moderate, or high [25]. The included studies were additionally assessed using a checklist appropriate to the study design. The Physiotherapy Evidence Database (PEDro) scale was used for the randomized controlled trials (RCT) or non-randomized controlled studies (NRS) [26]. PEDro scores range from 0–10 (a greater score indicates higher quality), with 6 as the cutoff score for high-quality studies [27]. PEDro scores were extracted from the official PEDro database [26]. Studies missing from the PEDro database were evaluated by two independent reviewers (CS & SK). The STROBE (Strengthening The Reporting of Observational Studies in Epidemiology) checklist was used to assess the quality of reporting for the observational studies [28]. The STROBE checklist consists of 32 items within six general sections with a maximum score of 32. A study with a STROBE score of 16 or above was considered to have a good methodological quality [5]. A funnel plot analysis evaluated the publication bias.

The publication characteristics (author(s), publication year, region), sample characteristics (sample size, age), and study characteristics (fall prevalence, the number of fallers, number of falls, and fall tracking approaches) were extracted by two reviewers (CS & SK). For each study, the annual fall prevalence was calculated as the ratio of the number of fallers to the sample size for either the AD or MCI group throughout a 12-month period [15]. A faller was defined as a person who experienced at least one fall during the 12 months. The average number of falls was defined as the total number of falls divided by the *sample size* within either group for each study.

### Meta-Analyses and between-Group Comparisons

The number of fallers, number of falls, sample size, and standard error (SE) values were extracted to calculate the pooled fall prevalence and average number of falls over a 12-month fall tracking duration. Extracted data were entered into Review Manager (RevMan) 5.3 software (Nordic Cochrane Centre, Denmark) and meta-analyzed using a random-effects model with the inverse variance weight. The 95% confidence intervals (CI) and 95% prediction intervals were estimated for the outcome measures. The meta-analyses were conducted separately for AD and MCI groups, and the results were presented using forest plots.

The annual fall prevalence was compared between AD and MCI groups using the *χ*^2^ test. To ensure the robustness of the comparison, a *Z*-score test was also utilized to compare the fall prevalence between groups. The significance level was set at *p* ≤ 0.05.

## RESULTS

### Study Selection

**Figure 1.**
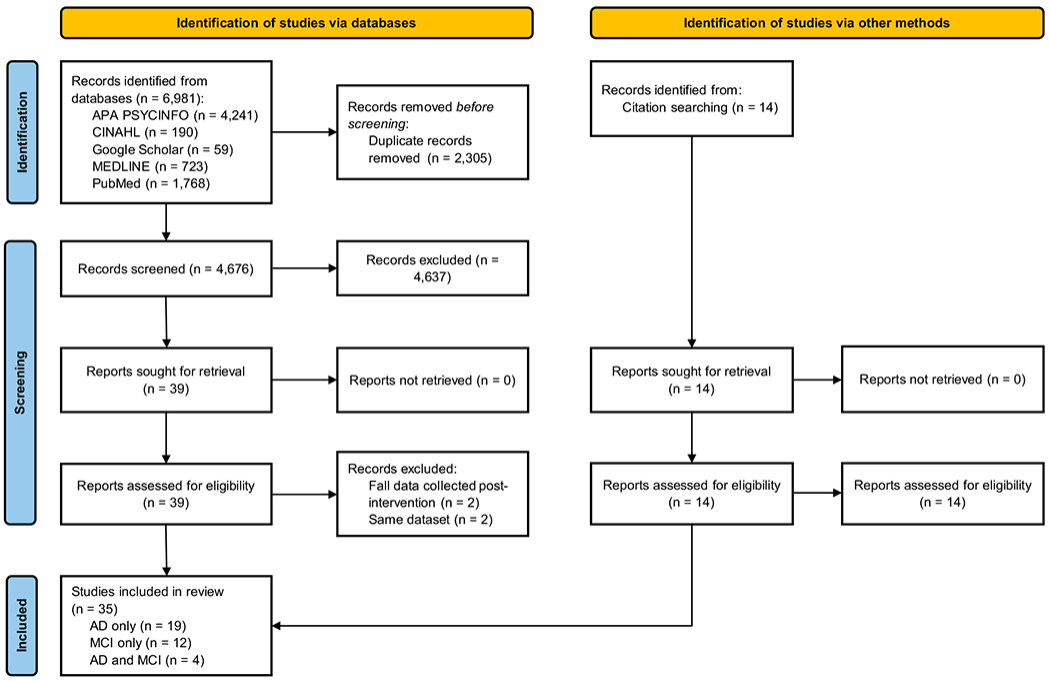
The Preferred Reporting Items for Systematic Reviews and Meta-Analyses (PRISMA) flow diagram showing the study selection process. The 35 included studies used participants with Alzheimer’s disease (AD), mild cognitive impairment (MCI), or both AD and MCI.

The initial literature search identified 6995 studies including 2305 duplicates. Further screening based on title and abstract removed 4676 more articles (Figure 1). Next, the remaining studies were screened based on the inclusion and exclusion criteria, and finally, 35 studies were deemed eligible for this meta-analysis.

### Characteristics of the Included Studies

Nineteen studies included older adults with AD only [29–47], 12 studies included older adults with MCI only [12,48–58], and four studies included both AD and MCI participants [14,59–61] (Table 1). The participants in the AD studies had a clinical diagnosis of AD. For MCI, the cognitive status of the participants was determined by various cognition assessment tools (e.g., the Mini-Mental State Exam [62], Montreal Cognitive Assessment [63], Frontal Assessment Battery [64], etc.). The average participant age spanned from 65 [34] to 86.6 [43] years for the AD participants, and from 69.5 [51] to 81.1 [55] years for MCI participants. All included studies tracked fall prevalence or the number of fallers during a 12-month period (either retrospectively or prospectively). The sample size varied significantly, from 14 [40] to 2490 [44] for AD studies and from 16 [56] to 938 [49] for MCI studies. The number of falls over a 12-month duration was available for five MCI studies [12,14,52,53,59] and nine AD studies [14,31–33,35,40,46,47,59]. Fall data were obtained primarily via participant self-report and were often verified by caregivers and/or physicians (Table 1). For a few studies, more specific tools were utilized to track falls including monthly calendars [12,32], phone calls [12,34], and diaries [50] (Table 1).

### Quality Assessment

**Figure 2.**
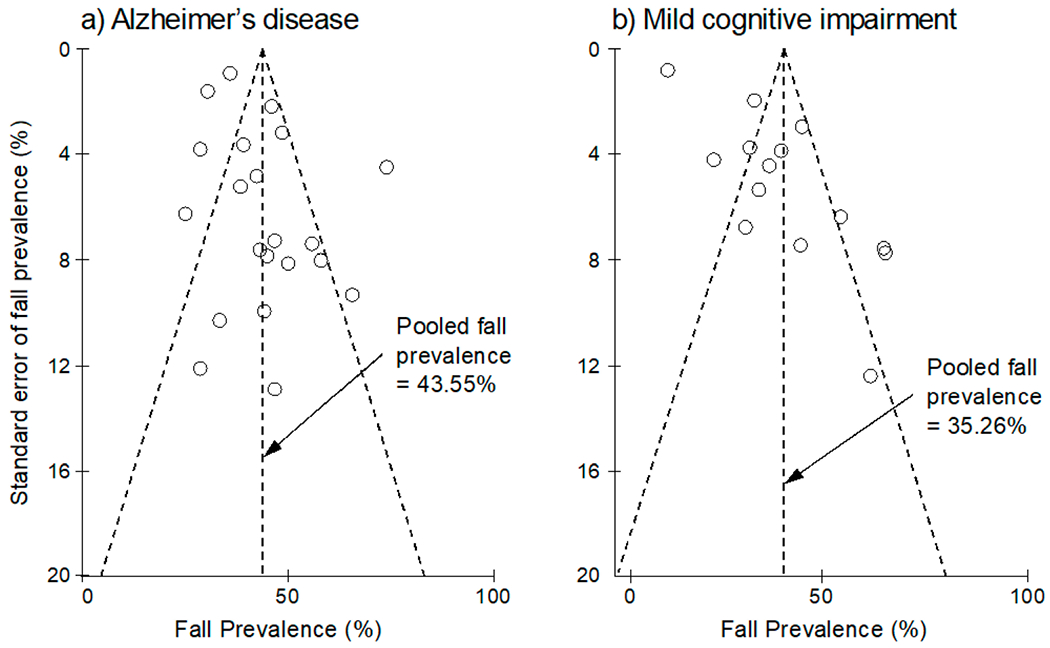
The funnel plots for the a) Alzheimer’s disease (AD) and b) mild cognitive impairment (MCI) groups. The Y-axis shows the standard error of fall prevalence (%), and the X-axis indicates the fall prevalence (%). Each of the open circles represents the observed point estimate for an individual study. The vertical dashed line represents the annual pooled fall prevalence for AD (21 studies) and MCI (14 studies). Among the 23 studies related to AD fall risk, 21 reported the annual fall prevalence for AD. Fourteen out of 16 studies included in this meta-analysis reported fall prevalence data for MCI. All observed point estimates are almost symmetrical around the pooled fall prevalence, and most studies are inside the inverted funnel region, indicating a low publication bias.

Using GRADE, the quality of the evidence was assessed to be low to very low for the included outcomes (Table 2). The mean PEDro score for the RCT and NRS was 4.0 ± 1.8 (Appendix 2), and the mean STROBE score for the observational studies was 19.3 ± 2.9 (Appendix 3). Considering the PEDro and STROBE cutoff points, the average scores showed a fair to good quality for the included studies. The funnel plot indicated a low publication bias for the fall prevalence meta-analyses (Figure 2).

### Meta-Analysis: Fall Prevalence

**Figure 3.**
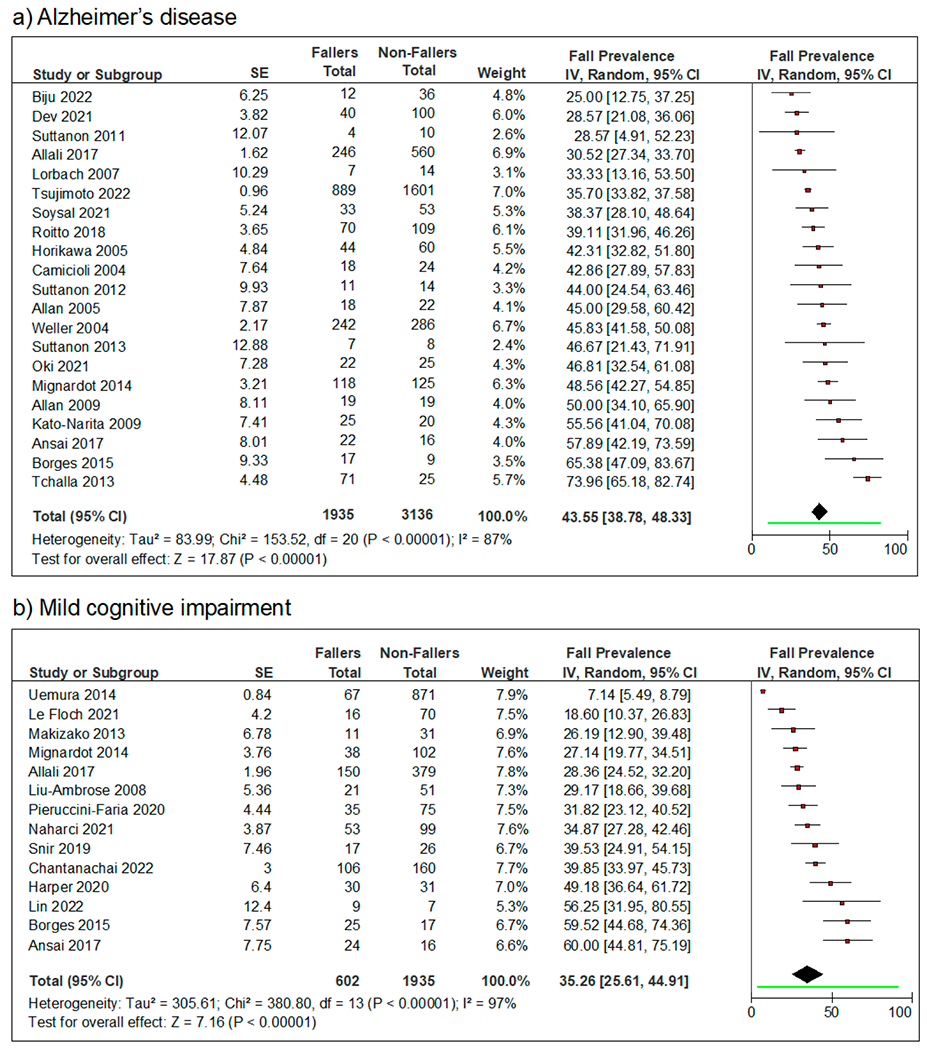
Forest plots of the fall prevalence over 12 months of older adults with (**a**) Alzheimer’s disease and (**b**) mild cognitive impairment. The random-effects model with inverse variance weights is used to estimate the pooled fall prevalence for each group. The line segment beneath the diamond indicates the 95% prediction interval for each meta-analysis.

The 31 included studies in the fall prevalence meta-analysis enrolled a total of 7608 participants. Among the included 5071 people with AD, 1935 people were fallers. The fall prevalence for people with AD varied considerably from 25% [32] to 73.96% [43], and the pooled annual fall prevalence was 43.55% (95% CI = [38.78%, 48.33%]) (Figure 3a). For MCI, 2537 participants were enrolled in the selected studies. Among them, 602 people were fallers. The MCI fall prevalence also varied drastically from 7.14% [49] to 60% [59] between studies, and the pooled fall prevalence was 35.26% (95% CI = [25.61%, 44.91%]) (Figure 3b).

### Meta-Analysis: Number of Falls

**Figure 4.**
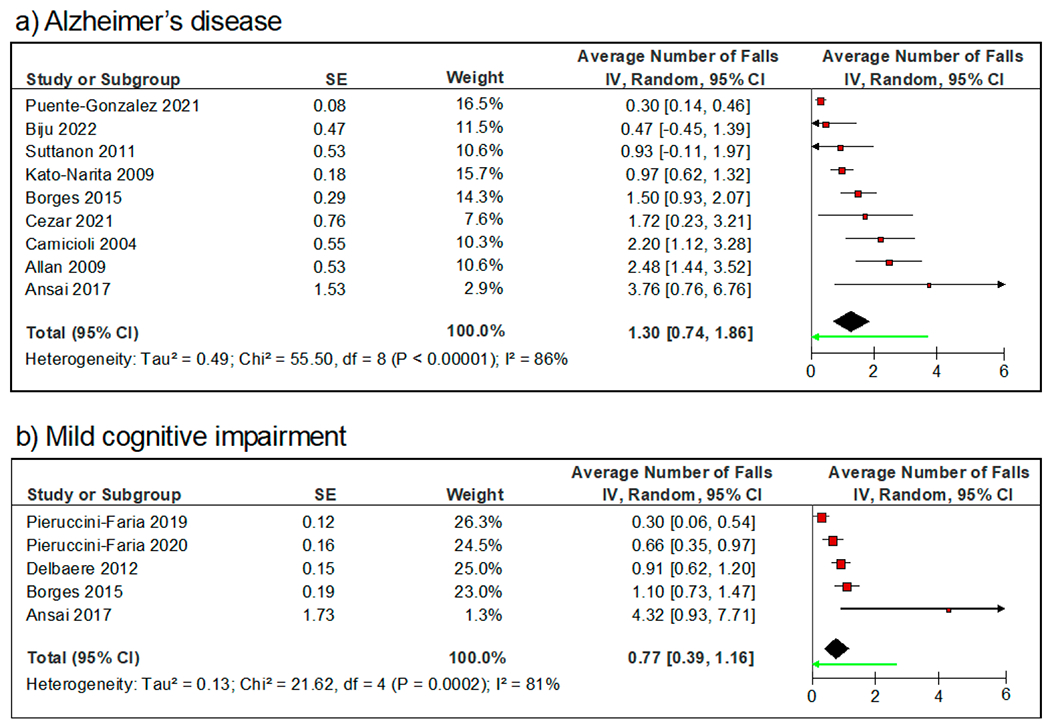
Forest plots of the annual average number of falls over 12 months among older adults with (**a**) Alzheimer’s disease and (**b**) mild cognitive impairment. The random-effects model with inverse variance weights is used to estimate the pooled number of falls for each group.

The cumulative annual average number of falls was 1.30/person (95% CI = [0.74, 1.86]) for AD (Figure 4a) and 0.77/person (95% CI = [0.39, 1.16]) for MCI (Figure 4b). The average number of falls in people with AD varied from 0.30/person [46] to 3.76/person [59] (Figure 4a). For MCI, the average number of falls varied between 0.30/person [53] and 4.32/person [59] (Figure 4b).

### Between-Group Comparison

The between-group (AD vs MCI) comparison of the pooled annual fall prevalence revealed a 1.24-fold fall prevalence in older adults with AD relative to those with MCI (43.55% vs 35.26%). Additionally, the *χ*^2^ test indicated that the pooled fall prevalence is significantly higher in people with AD than in people with MCI (*χ*^2^ = 158.403, *p* < 0.001). The *Z*-score test confirmed that more people with AD fall annually compared to their MCI counterparts (*Z* = 12.58, *p* < 0.001). The participants with AD also exhibited a higher average number of falls than their MCI peers (1.30 vs 0.77 falls/person/year).

## DISCUSSION

Although it has been overall recognized that older adults with AD or MCI have a higher fall prevalence than their cognitively healthy counterparts [14,31], it remains inconclusive whether the fall risk is similar or different between people with AD and people with MCI. The present meta-analysis aimed to determine the fall risk for people with AD and MCI separately and then compare the fall risks between the two groups. The fall prevalences derived in our present analysis were similar to those from previous meta-analyses. For example, one recent meta-analysis reported an annual fall prevalence of 34% among people with MCI [66], and another one indicated that the annual fall prevalence for people with AD is around 44% [15]. The findings supported the hypothesis that people with AD would have a higher fall risk than people with MCI. More specifically, the meta-analyses indicated that the composite annual fall prevalence and number of falls are 43.55% and 1.30 falls/person for people with AD and 35.26% and 0.77 falls/person for people with MCI, respectively. The annual fall risk is significantly higher among people with AD than those with MCI.

The discoveries of the higher fall risk in people with AD or MCI than in older adults without dementia are in line with previous findings [14,31,43]. In detail, the annual fall prevalence for people with AD (43.55%) or MCI (35.26%) is greater than that for older adults with intact cognition (26.5%) [5]. On average, people with AD or MCI experience more falls than cognitively healthy older individuals over a year (1.30 falls/person/year for AD and 0.77 falls/person/year for MCI vs 0.21 falls/person/year for healthy older adults [67]).

Dementia is associated with poor balance [48], mobility deficits [22], and reduced physical activity and functional status [68], which accounts for the higher fall risk in people with AD or MCI than in cognitively healthy individuals. Compromised balance and gait, mobility decline, history of previous falls, and impaired cognition are among the common fall-related risk factors for people with AD [19,69,70]. It has been reported that the relationship between gait impairment and falls is modulated by multisensory impairments that can lead to cognitive decline [71,72]. Namely, the more severe the cognitive impairments, the stronger the correlation between falls and cognitive deficits [73]. This relationship not only explains why people with dementia exhibit a higher fall risk than their cognitively intact peers, but also the greater fall risk among people with AD than those with MCI since AD is a more advanced dementia stage than MCI. Another possible contributor to the positive correlation between the severity of cognitive impairment and fall risk in older adults could be the side effects of taking medication(s) used to treat dementia. The potential side effects include dizziness, postural instability, confusion, difficulty in coordinating body movements, and decreased blood pressure, which can all increase the fall risk. One previous study also reported that polypharmacy is very common in people with cognitive impairment and that a higher number of pharmaceuticals being taken at one time is associated with a higher risk of falls and hospital admissions [74].

In addition to cognitive impairment, which compromises gait functions and increases fall risk, various other factors contribute to a heightened fall prevalence among individuals with AD and MCI. Previous studies have suggested that individuals with AD or MCI show poorer tactile discrimination, peripheral nerve compound muscle action potential amplitudes, and vibration sensation compared to cognitively healthy older adults [75]. Furthermore, the proprioception system plays a vital role in preventing falls [76–79]. Neurodegenerative changes and cognitive decline can impair proprioceptive functions in people with dementia. Thus, people with AD and MCI may have altered gaits, mobility deficits, and balance dysfunctions, which are also associated with somatosensory impairments and can further elevate the fall risk among these individuals [80,81].

This is the first study comparing the fall risk between people with AD and MCI. The analyses suggest that people with AD exhibit a significantly higher annual fall prevalence than people with MCI. This information could assist with making policies targeting fall prevention in people with dementia and better allocate fall prevention resources between people with AD and MCI. Another strength of this analysis is the inclusion of the number of falls as a fall risk measurement. As anticipated, people with AD show a substantially higher number of falls annually compared to individuals with MCI. Specifically, people with AD experience almost twice the number of falls as their MCI counterparts (1.30 vs 0.77 falls/person). This valuable information not only reinforces the higher fall risk in people with AD compared to MCI but also furnishes clinically meaningful information for clinicians and researchers to estimate the number of falls in people with AD or MCI at the population level. Collectively, the annual fall prevalence and fall frequency deepen the understanding of the fall risk in these two populations, facilitating the development of targeted fall prevention strategies for these individuals. The communities and organizations involved in supporting people with dementia can also benefit from the findings of this meta-analysis and establish relevant regulations.

The ideal approach to comprehensively and accurately determine the fall risk in people with AD or MCI would be to conduct a large-scale epidemiologic study across regions and races similar to those that have been performed for the general older adult population [82,83]. Studies of this design will avoid bias and heterogeneity between individual studies regarding data collection approaches and results reporting methods. However, before epidemiologic studies can occur, the best alternative would be to meta-analyze the individual published studies. In the current meta-analysis, the random-effects model was adopted, which could reduce the effects of the study variations on the results. In addition, the assigned weight to each study based on the sample size could also avoid the issue of biased results if a simple arithmetic mean was used to represent the overall fall measurements [84].

As indicated by the results, people with AD or MCI face a high risk of falling, which could lead to fall-related injuries. It is critically important to develop effective treatments to lower the fall risk for these populations. The information from this meta-analysis could guide different stakeholders such as policymakers to better distribute fall prevention resources among people with AD or MCI. It can also assist researchers and clinicians with developing modalities to assess fall risk, identifying individuals with an elevated fall risk, and establishing treatment strategies to lower the fall risk for these populations.

## LMITATIONS

This meta-analysis has limitations. First, the present study only considers the annual fall prevalence. It, therefore, remains unknown how the findings can be generalized to other fall tracking periods. Second, other fall-related metrics such as recurrent fallers, injured falls, and specific injuries were not considered in this meta-analysis. The inclusion of these measures could provide additional useful information to better understand the fall risk in people with AD or MCI and how the dementia stage impacts the fall risk. Third, due to the small sample size of studies included in this meta-analysis, the characteristics of the study, such as the region, participant race/ethnicity, sample size, etc., were highly diverse and resulted in a high heterogeneity of the meta-analyses, as indicated by the large *I*^2^ values. Fourth, the fall data were mainly collected based on monthly fall calendars and caregivers’ or nurses’ fall reports. This reporting method might be biased and subjective, and it could compromise the accuracy of fall records. Last, this meta-analysis was not prospectively registered with the International Prospective Register of Systematic Reviews. To the best of our knowledge, this is the first of its kind to compare the fall risk between people with AD and MCI. Thus, there is minimal risk of repeating a review on this topic. More high-quality and well-designed studies are needed to address these limitations.

## CONCLUSIONS

In conclusion, this meta-analysis revealed that the annual fall prevalence and number of falls are higher in older adults with AD than in their MCI counterparts. Therefore, the fall-related metrics should be reported separately between people with AD and MCI. The findings enrich the knowledge of the fall risk among people with dementia. The information derived from this study could provide a valuable reference for developing and implementing fall prevention programs targeting these populations. A reduction in fall risk would profoundly impact people with dementia, their caregivers and family members, and the healthcare system.

## Figures and Tables

**Table 1.** Summary of 35 included studies. Among them, 19 enrolled people with Alzheimer’s disease (AD) only, 12 studies involved people with mild cognitive impairment (MCI) only, and four studies included both AD and MCI.

Study	Country	Study Design	Fall tracking tools	Sample size	Participant mean age (years)
***Studies enrolling people with AD only***					
Allan 2009 [31]	UK	OBS	Self-report (−)	38	79
Allan 2005 [29]	UK	OBS	Self-report (−)	40	78.6
Biju 2022 [32]	US	OBS	Self-report monthly calendars (+)	48	74.9
Camicioli 2004 [33]	Canada	OBS	Self-report verified by staff (+)	42	82.6
Cezar 2021 [47]	Brazil	RCT	Caregiver report (−)	35	79.3
Dev 2021 [34]	Pakistan	OBS	Self-report monthly phone calls (+)	140	65
Horikawa 2005 [30]	Japan	OBS	Caregiver report (+)	104	74.1
Kato-Narita 2009 [35]	Brazil	NRS	Self-report verified by caregiver (−)	45	80
Lorbach 2007 [36]	Australia	OBS	Self-report (−)	21	79.3
Oki 2021 [37]	Japan	OBS	Caregiver report (−)	47	80.9
Puente-Gonzalez 2021 [46]	Spain	RCT	Self-report verified by caregiver (−)	72	76.7
Roitto 2018 [38]	Finland	RCT	Caregiver report (−)	179	77.6
Soysal 2021 [39]	Turkey	OBS	Self-report (−)	86	81
Suttanon 2012 [42]	Australia	NRS	Self-report verified by caregiver (−)	25	81
Suttanon 2011 [40]	Australia	OBS	Self-report verified by caregiver (−)	14	79.6
Suttanon 2013 [65]	Australia	NRS	Self-report verified by caregiver (+)	15	80.9
Tchalla 2013 [43]	France	RCT	Caregiver and/or physician report (−)	96	86.6
Tsujimoto 2022 [44]	Japan	NRS	Self-report verified by caregiver (−)	2,490	79
Weller 2004 [45]	Canada	OBS	Self-report (−)	528	83.2
***Studies enrolling people with MCI only***					
Chantanachai 2022 [50]	Australia	OBS	Self-report monthly diaries (+)	266	78.8
Delbaere 2012 [12] *	Australia	NRS	Self-report monthly diaries with follow-up phone calls (+)	77	77.5
Le Floch 2021 [54]	France	OBS	Self-report interviews (−)	86	70.9
Lin 2022 [56]	Taiwan	NRS	Self-report (−)	16	NR
Liu-Ambrose 2018 [51]	Canada	NRS	Physician report (−)	72	69.5
Harper 2020 [55]	Australia	OBS	Self-report (−)	61	81.1
Makizako 2013 [48]	Japan	RCT	Self-report interviews (+)	42	75.6
Naharci 2021 [57]	Turkey	NRS	Self-report (−)	152	80.1
Pieruccini-Faria 2019 [53]	Canada	NRS	Self-report (−)	52	72
Pieruccini-Faria 2020 [52]	Canada	OBS	Self-report (−)	110	74.3
Snir 2019 [58]	Canada	OBS	Self-report interviews (−)	43	74.5
Uemura 2014 [49]	Japan	OBS	Self-report (−)	938	71.9
***Studies enrolling people with AD and MCI***					
Allali 2017 [60]	Australia, Belgium, France, India, Luxembourg, Switzerland, US	OBS	Self-report (−)	806/529 ^$^	76.5
Ansai 2017 [59]	Brazil	OBS	Self-report verified by caregiver (−)	38/40 ^$^	76.7
Borges 2015 [14]	Brazil	OBS	Self-report verified by caregiver (−)	26/42 ^$^	75
Mignardot 2014 [61]	France	OBS	Self-report questionnaire (−)	243/140 ^$^	77.2

(−): Fall data were collected retrospectively. (+): Fall data were collected prospectively.

$: Sample size for AD/sample size for MCI.

OBS = Observational Study. RCT = Randomized Control Trial. NRS = Non-randomized Controlled Study. UK = United Kingdom. US = United States. NR = Not reported.

**Table 2.** The Grading of Recommendations, Assessment, Development and Evaluation (GRADE) assessment on the quality of the evidence at the outcome level.

Study information		Certainty assessment					Sample size	Effect	Quality of the evidence
No. of studies	Study design	Risk of bias	Inconsistency	Indirectness	Imprecision	Publication bias		Absolute (95% confidence interval)	
Fall prevalence—Alzheimer’s disease									
21	RCT	S ^a^	S ^b^	NS	NS	NS	*N* = 5071	43.55% fall prevalence (38.78 lower to 48.33 higher)	⊕⊕○○
	OBS & NRS	S ^a^	S ^b^	NS	NS	NS			⊕○○○
Fall prevalence—Mild cognitive impairment									
12	RCT	S ^a^	S ^b^	NS	NS	NS	*N* = 2537	35.26% fall prevalence (25.61 lower to 44.91 higher)	⊕⊕○○
	OBS & NRS	S ^a^	S ^b^	NS	NS	NS			⊕○○○
Number of falls—Alzheimer’s disease									
9	RCT	S ^a^	S ^b^	NS	NS	NS	*N* = 358	1.30 number of falls (0.74 lower to 1.86 higher)	⊕⊕○○
	OBS & NRS	S ^a^	S ^b^	NS	NS	NS			⊕○○○
Number of falls—Mild cognitive impairment									
5	RCT	S ^a^	S ^b^	NS	S ^c^	NS	*N* = 321	0.77 number of falls (0.39 lower to 1.16 higher)	⊕○○○
	OBS & NRS	S ^a^	S ^b^	NS	S ^c^	NS			⊕○○○

Quality of the evidence legend: ⊕○○○: Very low; ⊕⊕○○: Low; ⊕⊕⊕○: Moderate; ⊕⊕⊕⊕: High. RCT: Randomized Control Trial. OBS: Observational Study. NRS: Non-randomized Controlled Study. S: Serious. NS: Not Serious. Explanations:

a Majority of studies relied on self-report data.

b High heterogeneity amongst studies as evidenced by *χ*^2^ and *I*^2^.

c Small number of studies included.

## Data Availability

The dataset of the study is available from the authors upon reasonable request.

## APPENDICES

**Appendix 1.** The keywords and strategies used to search the five common databases, and the number of articles yielded from each search.

Database	Search Terms	Articles found
APA PSYCINFO	Search 1: “Alzheimer’s disease” AND “Accidental falls”	96
	Search 2: “Accidental falls”	3727
	Search 3: “Falls” AND “Alzheimer’s disease”	297
	Search 4: DE “Alzheimer’s disease” AND (DE “Falls” OR DE “Accidents”)	51
	Search 5: “MCI” AND “Falls”	70

CINAHL	Search 1: (MH “Alzheimer’s disease”) AND (MH “Accidental falls”)	190

Google Scholar	Search 1: “Alzheimer’s disease” AND “Fall prevalence”	59

MEDLINE	Search 1: “Falls” AND “Alzheimer’s disease”	723

PUBMED	Search 1: “Alzheimer’s disease” AND “Falls”	607
	Search 2: “Alzheimer’s disease” AND “Falls” NOT “Parkinson”	509
	Search 3: “Alzheimer’s disease” AND “Falls” AND “MCI”	44
	Search 4: “Accidental falls” AND “Alzheimer’s disease”	153
	Search 5: “Alzheimer’s disease” AND “Fall”	455

**Appendix 2.** The Physiotherapy Evidence Database (PEDro) scores of included studies adopting a randomized controlled design or a non-randomized control design.

Item	Cezar 2021 [47]	Delbaere 2012 [12]	Kato-Narita 2009 [35]	Lin 2022 [56]	Liu-Ambrose 2008 [51]	Makizako 2013 [48]	Naharci 2021 [57]	Pieruccini-Faria 2019 [53]	Puente-Gonzalez 2021 [46]	Roitto 2018 [38]	Suttanon 2013 [65]	Suttanon 2012 [42]	Tchalla 2013 [43]	Tsujimoto 2022 [44]
1. Eligibility criteria*	1	1	1	1	1	1	1	1	1	1	1	1	1	1
2. Random allocation	1	0	0	0	0	1	0	0	0	1	0	0	1	0
3. Allocation concealment	1	0	0	0	0	0	0	0	1	0	0	0	0	0
4. Baseline comparability	1	0	1	1	0	0	1	1	1	1	0	0	1	0
5. Blind participants	0	0	0	0	0	0	0	0	0	0	0	0	0	0
6. Blind therapists	0	0	0	0	0	0	0	1	1	0	0	0	0	0
7. Blind assessors	1	0	0	0	0	0	0	0	1	0	0	0	0	0
8. Adequate follow-up	1	1	0	0	1	1	0	0	1	0	1	0	1	0
9. Intention-to-treat	1	0	0	1	0	0	0	0	0	0	0	0	0	0
10. Between group comparisons	1	1	1	1	1	1	1	1	1	1	1	1	1	1
11. Point measures, measures of variability	1	1	1	1	1	1	0	1	1	1	1	1	1	1
Total PEDro score	8	3	3	5	3	4	3	4	7	4	3	2	5	2

1: yes, 0: no.

* Not used to calculate the total PEDro score. Mean ± standard deviation score: 4.0 ± 1.8.

**Appendix 3.** The Strengthening the Reporting of Observational Studies in Epidemiology (STROBE) scores of included observational studies.

Study	Title and abstract (1.a, 1.b)*	Introduction (2, 3)	Methods (4–12)	Results (13–17)	Discussion (18–21)	Other information (22)	Total score
Allali 2017 [60]	2	2	8	5	3	1	21
Allan 2005 [29]	2	2	9	9	4	1	27
Allan 2009 [31]	1	2	6	8	3	0	20
Ansai 2017 [59]	2	2	7	3	3	1	18
Biju 2022 [32]	2	2	8	4	3	1	20
Borges 2015 [14]	1	2	6	2	2	1	14
Camicioli 2004 [33]	2	1	7	8	3	0	21
Chantanachai 2022 [50]	2	2	6	6	3	1	20
Dev 2021 [34]	2	1	5	4	2	1	15
Harper 2020 [55]	2	2	9	4	4	0	21
Horikawa 2005 [30]	2	1	5	6	3	0	17
Le Floch 2021 [54]	1	2	7	3	4	0	17
Lorbach 2007 [36]	1	2	8	3	4	1	19
Mignardot 2014 [61]	2	2	8	3	3	0	18
Oki 2021 [37]	2	2	7	6	4	1	22
Pieruccini-Faria 2020 [52]	2	2	8	6	3	0	21
Snir 2019 [58]	1	2	9	5	4	0	21
Soysal 2021 [39]	1	2	8	5	3	1	20
Suttanon 2011 [40]	1	2	5	5	4	0	17
Uemura 2014 [49]	2	2	8	5	3	1	21
Weller 2004 [45]	1	1	5	5	3	0	15

* The numbers inside the parentheses are the item numbers included in each of the six STROBE sections. Mean ± standard deviation score: 19.3 ± 2.9.

## ABBREVIATIONS

AD: Alzheimer’s disease
CI: confidence interval
GRADE: Grading of Recommendations, Assessment, Development, and Evaluation
MCI: mild cognitive impairment
NRS: non-randomized studies
PEDro: Physiotherapy Evidence Database
PRISMA: Preferred Reporting Items for Systematic Reviews and Meta-Analyses
RCT: randomized controlled trials
RevMan: Review Manager
SE: standard error
STROBE: Strengthening The Reporting of Observational Studies in Epidemiology
